# Unpacking the teaching–research nexus: a phenomenographic analysis of student experiences in a medical university

**DOI:** 10.1186/s12909-026-10653-5

**Published:** 2026-10-21

**Authors:** Madelen Lek, Terese Stenfors, Per J. Palmgren

**Affiliations:** Department of Learning, Informatics, Management and Ethics, Karolinska Institutet, Stockholm, SE-171 77 Sweden

**Keywords:** Teaching–research nexus, Phenomenography, Students’ perceptions, Qualitative research, Higher education, Faculty

## Abstract

**Background:**

The integration of research and teaching in higher education has been widely discussed, with growing emphasis on fostering research-based learning environments. Within medical and health professions education, this integration is particularly critical, as research literacy and evidence‑based practice form core professional competencies. The concept of the teaching–research nexus posits that research-active faculty enhance student learning by introducing current knowledge and inquiry-based practices into the classroom. This not only deepens students’ subject understanding but also cultivates critical thinking and problem-solving skills. Despite its significance, there is a lack of studies exploring students’ perceptions of this nexus. This study addresses the identified need for qualitative insights and aims to explore what students understand as research and how the teaching–research nexus is enacted in their educational context.

**Methods:**

A phenomenographic design was employed to investigate students’ conceptions of research and how the teaching-research nexus is enacted in their educational context. Semi-structured interviews were conducted with 20 students from five academic programmes: Twelve enrolled in first-cycle health professions degrees and 8 in international master’s programmes in non-clinical disciplines. The analysis followed Dahlgren and Fallsberg’s seven steps of phenomenographic analysis.

**Results:**

Analysis resulted in four distinct categories of understanding research, presented in an order that reflects increasing complexity: (1) “Research is a spark of curiosity,” (2) “Research is exploring a knowledge gap,” (3) “Research is examining and providing new knowledge,” and (4) “Research is a continuous practice.” These categories were structured along three themes of expanding awareness: students’ own engagement with research, their perceptions of faculty as research-active educators, and the nature of teaching and learning activities. Together, these elements form the study’s outcome space, illustrating variation in students’ understandings, from passive exposure to active, reflective engagement in research-informed learning.

**Conclusions:**

Findings suggest that aligning pedagogical strategies with students’ current understanding of research can facilitate their transition from passive recipients to active participants in research. Faculty members play a pivotal role as scholarly role models, shaping students’ academic identity and reinforcing the dynamic nature of the teaching–research nexus.

**Supplementary Information:**

The online version contains supplementary material available at https://doi.org/10.1186/s12909-026-10653-5.

## Background

Universities have long shaped how knowledge is created, shared, and applied across society, and they continue to influence both academic and professional fields in significant ways. Their central role in generating and disseminating knowledge has been widely recognised in the literature [1]. Today, higher education faces a wide range of challenges. In medical and health professions education, these challenges are amplified by rapid scientific development and the expectation that graduates engage in evidence-based clinical and academic decision-making. One major topic of discussion includes the nature and purpose of higher education and the status and role of research in modern universities [2]. Universities are often regarded as key institutions of the knowledge society and are established on the principles of research training and related scientific knowledge continuity [3]. This discussion has also been prominent within medical and health professions education, where research‑integrated teaching is viewed as essential for preparing future practitioners to engage in evidence‑based decision‑making and to contribute to knowledge production in rapidly evolving clinical and educational environments [4].

Research-based education is shaped by the Humboldt ideal of a university where research and teaching take place side by side. It is also anchored in Newman’s view of useful and liberal knowledge, where students are supported to become a community of thinkers [5]. This relationship between research and teaching has been widely examined in higher education, often framed through the concept of the research–teaching nexus, which describes how these activities may be connected. Neumann [6] further showed that academics perceive this nexus in different directions, with research shaping teaching, teaching shaping research, or both, highlighting that the relationship is not inherently unidirectional. Building on this discussion, the inverted concept of the teaching–research nexus, frequently linked to the Humboldtian model [7], reflects a growing recognition of the interconnected roles of research and teaching in academia. Boyer [8] broadened the concept of scholarship by arguing that teaching should be valued alongside research, integration, and application. Hattie and Marsh [9] challenged the assumption that research productivity and teaching effectiveness are inherently related, showing that research-active academics do not necessarily provide research-integrated teaching. Their work shifted attention towards understanding how research can be deliberately and meaningfully integrated into educational practice.

In this paper, we adopt the term teaching–research nexus to emphasise the pedagogical processes through which research becomes visible and meaningful for students. While the concept is often framed as the research–teaching nexus, our inversion reflects a deliberate focus on students’ learning experiences rather than on research productivity alone. This relationship has long been recognised as central in medical and health professions education, where students must learn to navigate, interpret, and apply research as part of their future clinical and professional roles [10].

By actively engaging in research, faculty bring cutting-edge knowledge and practical insights into the classroom, deepening students’ understanding of the subject matter [11]. The teaching–research nexus also enhances students’ critical thinking and problem-solving skills through involvement in research activities [12], offering valuable experiential learning for diverse career paths. Moreover, it supports faculty members’ professional growth by keeping them current in their fields and refining their teaching through research-informed practices. Within medical and health professions education, this interplay between teaching and research has been particularly emphasised, as evidence‑based practice forms a core professional competency and requires that students learn to appraise, interpret, and apply research as part of their clinical and academic development [13].

Work in higher education has also demonstrated that students encounter the teaching–research nexus in varied ways, with differences across institutional contexts and in relation to how actively they are engaged in research-related activities [14]. Research on students’ engagement with research‑related activities suggests that such involvement plays a key role in shaping how the nexus is understood [15]. Broader reviews have additionally highlighted that the relationship between research and teaching is multifaceted and shaped by local conditions, underscoring the importance of understanding how different actors experience this connection [16]. Although empirical interest in the teaching–research nexus has increased [17], relatively little is known about how students themselves understand the relationship between research and teaching, particularly within medical and health professions education [18].

Understanding variation in students’ conceptions and experiences of the teaching–research nexus from an emic perspective can help educators make research more visible and meaningful within the curriculum and support students’ engagement with research. With this aim, the present study explored what students understand as research and how the teaching–research nexus is enacted in their educational context.

## Methods

Phenomenography as a research approach was developed within higher education research and seeks to understand and describe the different ways in which phenomena in the world are understood [19, 20]. Yu et al. describe how phenomenography can be distinguished from other qualitative research approaches by its focus on the variation in which people can understand the same phenomenon [21]. In conducting this study, we adhered to the Consolidated Criteria for Reporting Qualitative Research (COREQ) guidelines [22] as well as specific quality considerations as deemed suitable when using a phenomenographic approach [23].

### Context of the study

The study was conducted at Karolinska Institutet, a large medical university in Sweden that offers a range of first (Bachelor) and second cycle (Master’s) programmes, all within the broader field of health care and medical science. As Karolinska Institutet is a single faculty medical university, all educational programmes, including those represented in this study (see Table 1) are situated within the Faculty of Medicine, positioning the study firmly within the context of medical and health professions education. For this study, five programmes were selected to capture a variety of perspectives. The selection included both health professions degrees and international master’s programmes in nonclinical subject areas. In the context of Swedish higher education, where the authors of this paper are based, research connection refers to the requirement that teaching should be grounded in or informed by current research. This definition is anchored in Swedish higher education legislation, which mandates that education must rest on a scientific basis and that a close relationship between research and teaching must be maintained [24]. The concept emphasises a reciprocal relationship between teaching and research, in which research informs teaching, and teaching, in turn, can influence research [25]. Research connection further involves the active inclusion of students in research processes, for example through project work, thesis writing, or participation in ongoing research activities. In this way, both students and educators benefit from the dynamic interplay between knowledge creation and knowledge dissemination. Consequently, throughout the study, the term research connection is treated as equivalent to the term teaching–research nexus.

**Table 1:** Characteristics of the research participants

Number of participants	Educational programme	Participant characteristics
8	Physiotherapy	Mean age 31.3 years (Median age 26.5) 9 female 2 male 1 non-binary
4	Speech and Language Pathology	
3	Master‘s programme in Bioentrepreneurship	Mean age 25.4 years (Median age 25.5) 4 female 4 male
2	Master’s programme in Health Informatics	
3	Master’s programme in Health Economics, Policy and Management	

### Data collection

A purposive sampling of courses and programmes was carried out to ensure a rich and diverse set of student perspectives. The selection considered variation across clinical and theoretical courses, short and long programmes, and courses offered early or late in the educational trajectory. Programme directors were consulted to help with the identification of courses where research integration was either particularly evident or notably limited, thereby enhancing the variation in the sample.

Sixteen first cycle (undergraduate or Bachelor´s-level) students were invited to participate; four declined the invitation. Nine second cycle (postgraduate or Masters-level) students were invited, all accepted, although one later cancelled.

An interview guide was developed for this project (see Supplementary Material) and, consistent with phenomenographic interview technique [26], the guide comprised three groups of open-ended questions designed to elicit students’ ways of understanding research through both conceptual reflections and descriptions of concrete educational experiences. The first explored students’ conceptions of research, the second explored their understanding of the teaching–research nexus, and the third invited participants to describe examples from their educational experiences illustrating how research was connected to teaching and learning activities. The interview guide functioned as a flexible support rather than a fixed sequence, and questions were asked in varying order and adapted to the flow of each conversation. Follow‑up probes, both listed in the interview guide and spontaneous open-ended questions, were used as well. This approach is consistent with phenomenographic interviewing, where the aim is to encourage participants to elaborate freely and to capture variation in how the phenomenon is understood. Participants were prompted to draw upon concrete educational experiences, and these descriptions informed the interpretation of the phenomenographic categories rather than constituting a separate analytical outcome [26]. Prompts were used throughout the interviews to encourage participants to elaborate on their experiences.

Two pilot interviews were conducted to assess the clarity and suitability of the interview process and the guide. As the pilot interviews indicated no need for revisions to either the procedure or the content of the interview guide, they were included in the final dataset. All participants were informed about the study and its aim, as well as their right to decline to answer any questions or withdraw from participating in the study. In addition, all participants gave consent to the interview before the interview started. In total, 20 interviews were conducted: 12 with students from first-cycle education and eight with students from second-cycle education. The sample size was guided by the concept of information power, and the research team assessed the interviews to provide sufficient richness to answer the research problem and support transferability within similar contexts [27].

The characteristics of the participants are presented in Table 1. The interviews with second-cycle students were conducted in English, while the remaining 12 interviews were conducted in Swedish. All interviews were conducted via Zoom and lasted between 29 and 51 min. PP conducted all interviews. Field notes were taken during and immediately after each interview to document observations and reflections. All interviews were video-recorded and transcribed verbatim.

### Analysis

The analysis was initially structured with reference to Dahlgren and Fallsberg’s seven-step procedure [28]. While this procedure offers a clear and practical structure, it does not encompass all aspects of more recent phenomenographic methodology. Therefore, the analytic work was also informed by contemporary phenomenographic principles, including the more explicit exploration of structural relationships between categories [29]. An overview of the seven steps is presented in Table 2.

**Table 2:** The seven steps of phenomenographic analysis according to Dahlgren and Fallsberg

1. Familiarisation	Reading through the interview transcripts to get a fresh impression of how the interview proceeded. In this initial phase, all data are given equal consideration.
2. Condensation	Identifying meaning units in the dialogue and marking these for the purpose of further scrutiny.
3. Comparison	Comparing the units regarding similarities and differences in meaning.
4. Grouping	Allocating meaning units expressing similar ways of understanding the phenomenon to the same category, and those expressing different ways of understanding to different categories.
5. Articulating	Capturing the core meaning of a certain category.
6. Labelling	Expressing the core meaning of the category. Steps 3–6 are repeated in an iterative procedure to make sure that the similarities within and differences between categories are discerned and formulated in a distinct way.
7. Contrasting	Comparing the categories through a contrastive procedure whereby the categories are described in terms of their individual meanings as well as in terms of what they do not comprise.

ML served as the analytic lead. In the familiarisation step, PP and ML read all transcripts in full, and TS read six of the transcripts to obtain an overall sense of students’ descriptions of the phenomenon. ML then re-read the complete dataset several times to deepen understanding and familiarisation before identifying and marking meaning units in NVivo during condensation. These meaning units were reduced to their essential content while keeping the students expressed ways of experiencing the phenomenon intact. The condensed units were then compared for similarities and differences. Initially, meaning units relating to students’ conceptions of research, the teaching–research nexus, and educational experiences were examined in relation to the study aim. Questions concerning students’ educational experiences were not analysed as an independent dataset but were interpreted alongside participants’ descriptions of research and the teaching–research nexus, thereby enriching the interpretation of the phenomenographic categories. As the iterative comparison and grouping progressed, these strands of meaning were considered together to construct a single outcome space. In phenomenography, the outcome space refers to the structured set of qualitatively different ways in which a phenomenon is understood, together with the logical relations between these ways. It presents the final configuration of categories that emerged from the analysis and shows how they relate to one another. This follows Åkerlind’s description of the outcome space as the organised representation of variation in ways of experiencing a phenomenon [29]. Rather than constructing separate categories for research and the teaching–research nexus, meaning units from both aspects were iteratively compared and interpreted together to identify qualitatively different ways in which students understood research. Preliminary groupings were iteratively refined during the subsequent steps of articulating and labelling. In the articulating step, ML began drafting the initial formulation of each category and exploring how the categories related to one another. Regular discussions with PP and TS served to question interpretations, confirm distinctions, and strengthen the analytic reasoning. This required repeatedly moving between individual transcripts, sets of meaning units, and early category structures to ensure that each emerging category was internally coherent and qualitatively distinct from the others. These discussions contributed to identifying the final set of descriptive categories representing the variation in students’ ways of perceiving the phenomenon. The labelling step involved naming each category in a way that succinctly captured its critical features. The themes of expanding awareness were constituted by comparing the categories in terms of similarities and differences in their individual meanings and structural relationships. Through this comparison, patterns of expanding awareness across categories were identified, which helped differentiate the structural relationships between categories. This iterative contrastive process continued until the categories were judged to be conceptually robust, qualitatively distinct, and collectively representative of the dataset, as well as illustrating the expanded awareness of the students [30].

### Research team and reflexivity

The multidisciplinary research team included two females and one male. ML is the programme director for one of the Master’s programmes at the university, from which three students participated. PJP and TS had no prior connection with any of the participants, nor are they involved in any of the programmes from which students were recruited. PJP and TS are medical education researchers with extensive expertise in qualitative research. All authors are faculty members at the university where the study took place. As university faculty, the concept of the teaching–research nexus was familiar to the researchers when the study began. However, none of the authors had previously conducted research specifically on this concept.

The trustworthiness of the study was strengthened through investigator triangulation. PJP and TS were involved in the early planning and design of the study, and ML joined the research team at the start of the analysis phase. All three researchers contributed to the analytic process and to the writing of the manuscript, allowing the phenomenon to be examined from multiple perspectives. To support dependability, the study design and procedures for data collection were documented in detail. The stability of the data interpretation was reinforced through repeated readings of the transcripts by the research team, followed by discussions until shared understandings were reached. Credibility was further enhanced by the active participation of ML, TS, and PJP in the analytic work and by the use of representative quotations to demonstrate the empirical grounding of each category. Throughout the analysis, continual comparisons were made between emerging subcategories and categories and the original transcripts. This iterative process, informed in part by the first author’s contextual understanding, also followed the criteria for quality in phenomenographic research suggested by Sin [23].

## Results

We explored students’ understanding of the phenomenon of research and how the teaching–research nexus is enacted in their educational context. The analysis shows a variation in the students’ perceptions, which can be categorised into four distinct ways of understanding research, differentiated by three themes of expanding awareness. These patterns reflect qualitatively different ways in which students at a medical university make sense of research and its role in their education. These categories represent the entire outcome space of the material; see Table 3. The subsequent analysis will detail these four categories, presented in terms of less to more complex perceptions of research.

**Table 3:** Outcome space of ways of understanding research with themes of expanding awareness

Themes of expanding awareness	Categories			
	1 - Research is a spark of curiosity	2 - Research is exploring a knowledge gap	3 - Research is examining and providing new knowledge	4 - Research is a continuous practice
*Students’ own engagement with research*	Students learn how to search for, handle and read research literature on a basic level.	Students learn how to use research as a point of reference and to assess the quality of research.	Students learn how to discuss the evidence base for different interventions and discover a discrepancy between theory and practice.	Students learn how to compare and contrast scientific literature and understand the value of keeping themselves updated in their field.
*Students’ perception of faculty as research-active educators*	Faculty add references to scientific literature in their lectures.	Faculty use the latest research in their teaching (sometimes their own) and talk about which research is relevant and reliable.	Faculty share the evidence base of their teaching by referring to their own research as well as others.	Faculty act as role models and experts in their field and invite students to join their research projects.
*Students’ experience of research-based teaching and learning activities (TLAs).*	TLAs focus on memorizing and repeating facts from literature.	TLAs focus on discussions of specific research literature	TLAs focus on discussions where students have to find their own evidence base.	TLAs focus on reflecting the student’s own knowledge through independent research reports.

### Research is a spark of curiosity

In this category, the students perceived research as passive, something they think about or are subjected to. Research in this category was generalised to something inherent or a mindset.

It’s about curiosity—wanting to understand the world, oneself, and how things work. *BSc 9*.

Students perceived research as the ability to find and read scientific literature on a subject they were interested in, or that was the focus of their course. They talked about getting tools or resources to handle research.

“The course I just attended was very research connected in the sense that it provided us with tools to handle research. In earlier courses, research was mostly cited to provide arguments or references were included in presentation slides.

BSc 1.

The actions were passive; to learn how to search bibliographic databases and understand how to read scientific literature. However, the students did not express any intention to assess the quality of the literature or to read more than what was required. The students also spoke about research as a way to pursue their interests.

The student engagement in research in this category was low. Students expected faculty to add lists of references in their presentations as a sign of the connection to research. However, the students mentioned that they are unlikely to go back to look at the references if they are not explicitly told to. One student perceived that the faculty’s role is to “implement their research knowledge” on the students, aligning with the passive perception of research in this category. Some students suggested that the credibility of the university and its faculty was more important for the perceived research quality than the credibility of the scientific literature recommended by the faculty.

The teaching and learning activities (TLAs) were of a more introductory or generalised nature. Getting introduced to the course literature and selected scientific literature sources was perceived to help students pursue their “spark of interest”. The TLAs mentioned by students included an introduction to information seeking from the university library, i.e. learning how to find and access scientific literature. Other examples mentioned by the students were reading scientific literature and writing a learning log or learning specific facts from literature and being able to repeat them in a written exam. The TLAs were assessed as more helpful if the faculty had pedagogical skills than if they were active researchers.

Ultimately, this category was about research as inquiry, and the students expected the answer to be provided to them by the faculty or other university staff, such as librarians. Research occurred at the moment the student got a spark of interest that started their thinking about the “why”. This can be compared to the next category, where research was seen as more activity-based, something that you do, to explore what you do not know of yet.

### Research is exploring a knowledge gap

In this category, the students moved beyond perceiving research as a passive activity and instead started to actively explore how to fill their experienced knowledge gap.

For me, it’s about solving problems. Sometimes the problem is simply a lack of knowledge—filling a knowledge gap or finding a treatment.

*BSc3*.

The students started to engage more and search for answers, and as one student put it: *“I think research*,* for me*,* is about seeking answers to a question—or several questions—that one might have.” MSc2.*

Research began to be perceived as something you could do, although the thought had not translated into doing it yourself. The students got accustomed to words like examining and testing, getting familiar with the “research language”. Some students started talking about finding solutions. As BSc5 stated, *“We have different solutions*,* but none that work particularly well. Research can involve testing existing solutions to see which one works best or understanding why they don’t work. But it can also mean trying to find a solution where none currently exists*”.

The students also perceived that research included testing previous findings and examining if the current knowledge is reasonable: *“It’s about examining what is reasonable*,* building on the findings of others*,* and then either challenging or strengthening those findings.” BSc1.*

Research literacy in this category was perceived as not only finding and being able to read scientific literature but also included evaluating the quality of the sources and being able to relate literature from the classroom to practical aspects of their studies and future work. More than just acquainting yourself with how to handle research information, this category was about understanding the “latest” research and integrating research as a point of reference in their studies.

The students not only perceived it as important that the faculty used references in their teaching activities, but also that the presented research was relevant and new. Faculty were expected to give the students the “latest” research and provide extra literature on interesting aspects. Students also relied on faculty to tell them which research was reliable and how they could keep updated in their field.

“The first thing that comes to mind is connecting real research to my class—even though we’re not researchers yet. Our teachers—our lecturers—are researchers, most of them at least.” BSc3.

TLAs mentioned in this category included discussing research with peers in, e.g. literature seminars or patient cases, where faculty distributed selected scientific literature for the students to read and discuss according to a set of questions. There was a right and wrong answer to these questions, and the students were expected to verify their answers with the supplied literature.

For example, we might be given a task or group assignment in class, and the professor provides some background information. Using that, we discuss—either in groups or individually—and come up with a method to solve the task with evidence from scientific literature. It’s a mix of personal thinking and research-based knowledge.

*MSc4*.

The following category moves even further, talking about research as working evidence-based or with a hypothesis.

### Research is examining and providing new knowledge

This category is perceived as more than actively exploring a knowledge gap and understanding research language. It is about the students examining and engaging in knowledge creation.

Research is about finding evidence for how things work. It’s also about discovering new treatments—testing whether something works, like whether it can help someone with chronic pain, for example.

*BSc6.*

Students continued to assess the quality of the scientific literature, and what was perceived to be reliable knowledge was given more importance. The students mentioned that they started seeing discrepancies in how theory and practice were aligned in their curriculum, as the methods from the classroom were not always used in the workplace. The students perceived that what was taught (both theoretically and practically) should be proven or evidence based. Along the same lines, the students mentioned testing (a hypothesis) and making conclusions proving that something worked as expected.

I think research involves creating a hypothesis, testing it, and then arriving at a conclusion—or developing a new hypothesis.

*BSc4.*

It should be evidence-based. What we talk about in education, or what we teach, should be connected to research.

*BSc6*.

The perception of research was strengthened by the perceived credibility of faculty, e.g. having faculty who were active researchers themselves. The students talked positively about how faculty integrated their own research, as long as it aligned with the subject taught. However, the students also perceived that the faculty needed to relate their research to other research and be able to share the common evidence base or conclusions of their taught subject.

TLAs mentioned by students revolved around integrating the given scientific literature with credible literature they found themselves. This could be in the form of discussions on certain topics or in minor research projects where the students learned how to use evidence-based methods. This was also mentioned in relation to work-integrated learning occasions.

### Research is a continuous practice

Research in this category was perceived as the “current truth“, and students were aware that research can be interpreted differently based on the speciality of the researcher who presents the research. Research was “alive” and changed to meet the current best knowledge, and they were aware of that. The students understood how to perform research projects and were able to relate research to their work “reality”.

Over time, my understanding has evolved. I’ve come to see that it’s not just the knowledge input that should be research-based—research itself is something living.”

*BSc 12*.

The students reflected that research was continuous and something you maintained throughout your life. Your knowledge and skills need to be updated even after graduation. The research connection was a loop (research informs practice that again informs the next iteration of research, and so on). There was credibility in knowing that there were continuous iterations of the research that formed the knowledge base of your studies.

To me, research connection means linking what you research, in the lab, with what happens in real life—especially in healthcare settings. It goes both ways: everyday practice should be grounded in research, and at the same time, real-life experiences can inform research.

*MSc3*.

Students in this category demonstrated an ability to compare and contrast findings from scientific literature, and they provided arguments for the relevance of the sources they used.

The students perceived faculty as role models and experts in their field. Faculty were expected to be open to discussions about how the best practices today might not be the same tomorrow, and that built trust in the process. More than that, faculty were open to inviting students to be part of research-led training in their research groups, either as part of their studies, e.g. when writing their degree projects, or as assistants in their extracurricular time.

TLAs in this category were perceived to encourage students to be part of the research conducted at the university. Longer, more independent research projects under the supervision of a researcher were mentioned, as well as study visits. The students were invited to reflect on their knowledge and experiences.

This final and most complex category includes the aspects from the three previous categories, moving from a passive, more descriptive way of describing research through understanding research as more active exploring, through testing and explaining, to reflecting on the iterative, living aspects of a constantly moving and evolving state of knowledge.

## Discussion

In this paper, we aimed to explore what students understand as research and how the teaching–research nexus is enacted in their educational context. The analysis showed a variation in students’ understandings of research, which was grouped into four categories: *research as a spark of curiosity*,* research as exploring a knowledge gap*,* research as examining and providing new knowledge*, and *research as a continuous practice.* This variation was further structured by three themes of expanding awareness: *students’ own engagement with research*,* their perception of faculty as research-active educators*, and *their experience of research-related TLAs.* Together, these categories and themes form the outcome space of the study and illustrate how some students have a less complex understanding of research, whilst others expressed a more complex and reflective participation in research-informed learning. This pattern highlights the dynamic nature of the teaching–research nexus as experienced by students in higher education. Students’ understandings of research and their experiences of research‑related teaching are linked. From a phenomenographic perspective, these findings should not be interpreted as indicating a causal relationship in which particular TLAs produce specific understandings of research. Rather, they reflect a relational view of learning, in which students’ understandings influence how they experience their educational environment, while those experiences simultaneously shape their understandings [31]. Taken together, these findings contribute to the medical education literature by illustrating how students at a medical university conceptualise research and perceive its integration within their teaching and learning activities.

The four categories identified in our analysis illustrate distinct ways in which students conceptualise the nexus in their education. The pattern reflects a deepening awareness of research not only as content but as a practice that is integral to academic and professional identity. These findings support the theoretical framing of the nexus as a relational construct, where students may express understandings ranging from peripheral engagement to more central participation in research processes.

To interpret the apparent alignment between students’ understandings of research and the TLAs they reported, it is important to acknowledge that several interpretations are possible. From a phenomenographic perspective, students do not experience TLAs as they objectively exist, but as they appear to them. This means that students with more complex understandings may be more likely to notice, value, or recall research‑related TLAs, whereas students with less complex understandings may overlook the same activities. Conversely, certain TLAs may genuinely support the development of more sophisticated understandings, but the present data cannot determine the direction of influence. The observed alignment should therefore be understood as a relational pattern between students’ ways of understanding research and the aspects of teaching they experience as meaningful, rather than as evidence of a direct causal relationship.

This pattern aligns with the work of Macheridis et al., who found that students at both research-intensive and teaching-intensive universities reported a progression in how they experienced the nexus over the course of their studies [14]. Their study also highlighted that students from research-intensive institutions perceived a stronger connection between teaching and research and were more likely to see this connection as relevant to their future professional lives. Our findings resonate with these observations, particularly in the more complex categories, where students expressed deepening engagement with research literature and tentatively described what might be interpreted as an emerging sense of involvement in the research process. These observations resonate with the typologies proposed by Visser-Wijnveen and colleagues, who emphasised the importance of students’ active involvement in research-related activities for fostering deeper learning [15]. In our study, students in the categories characterised by a more complex understanding described engaging in independent inquiry, evaluating evidence, and recognising faculty as research-active role models. These experiences reflect a shift from passive reception of knowledge to active participation in knowledge creation, which is central to the teaching–research nexus as we define it. Moreover, our findings support the argument made by Gros et al., who noted that while research-related competences are often embedded in course syllabi, students frequently perceive these as becoming meaningful only in the later stages of their education [32]. This suggests a need for earlier and more explicit integration of research practices into teaching, particularly through activities that scaffold students’ progression from basic engagement to more complex and reflective forms of inquiry.

The teaching–research nexus is not a fixed structure but a context-dependent and evolving relationship that requires careful pedagogical design. This view is supported by Elken et al.’s broader review, which highlights the need for more nuanced indicators and typologies to understand how research and teaching interact, including how different actors, such as students, experience this relationship [16]. Our results align with this perspective and reinforce the importance of integrating research and teaching in thoughtful ways.

We believe that our empirical work contributes to addressing a notable gap in the literature: the lack of research that explores students’ epistemological perspectives on the teaching–research nexus. Much of the existing research has focused on faculty perspectives or institutional strategies [32, 33], whereas our study provides a student-centred view of how the nexus is experienced and understood in practice. This perspective is essential for designing curricula that foster inquiry-driven learning environments where students see the relevance of research to their academic and professional development.

Our findings offer a more nuanced view of how research is experienced by students. Our results suggest that students experience the teaching research nexus not only through research‑based course content but also through engagement with research processes, reflection on the role of evidence in practice, and the early formation of a scholarly identity.

### Implications for practice

Building on these findings, it becomes clear that educators should consider students’ varying levels of research understanding when designing curricula and learning activities. By aligning teaching strategies with students’ different understandings of research, faculty can more effectively support the transition from passive reception of research content to active engagement in research practices. For example, integrating structured opportunities for literature analysis, evidence-based discussions, and small-scale research tasks early in the curriculum may help students progress toward more complex and reflective forms of inquiry. Faculty members also play a crucial role as role models; making their own research visible and accessible to students can strengthen the perceived relevance of research in education and foster a stronger sense of scholarly identity. In this way, the teaching–research nexus becomes a dynamic and evolving relationship shaped by students’ experiences, institutional contexts, and pedagogical practices.

### Methodological considerations

The choice of phenomenography was a suitable approach to capture the variation in students’ conceptions of the role of research in teaching. The iterative analysis followed the model proposed by Dahlgren and Fallsberg [28], with multiple researchers involved throughout the process. This collaborative approach enhances the study’s credibility. In addition, a diverse selection of programmes and courses and the inclusion of both international and national students contributed to a broad range of perspectives. However, all participants were recruited from the same higher education institution, which may limit the transferability of the findings. Interviews were conducted via Zoom, a format shown to be practical and conducive to participation in qualitative research [34]. Although this facilitated accessibility, the online setting may have affected conversational depth due to occasional challenges with non-verbal cues and connection quality. One of the researchers (ML) serves as the programme director for one of the participating programmes, which may have influenced the interpretation of the data. This was balanced by the involvement of two other researchers (PP and TS), who had no prior connection to the participants. Reflective notetaking as well as continuous discussions between the researchers were used to enhance trustworthiness.

### Future direction of research

Future research should continue to explore the teaching–research nexus from the student perspective, particularly through longitudinal and cross-disciplinary studies. Investigating how students’ perceptions evolve over time and across different academic fields could provide deeper insights into the conditions that support meaningful integration of research and teaching. Additionally, more research is needed on how institutional structures such as course design, assessment practices, and faculty development influence students’ engagement with research. Qualitative studies that capture students’ voices in diverse educational contexts will be especially valuable for informing evidence-based pedagogical strategies and policy development.

## Conclusions

The findings of this study suggest that students’ perceptions of research span from passive exposure to research content toward active engagement in research practices. The four categories identified, ranging from curiosity-driven views to conceptualisations of research as a continuous scholarly practice, reflect qualitatively different understandings of the enactment of the teaching–research nexus as expressed from the students’ own perspectives. These understandings relate to students’ own engagement, their recognition of faculty as research-active educators, and their understanding of the nature of teaching and learning activities. The findings highlight the importance of tailored teaching and learning activities to accommodate students’ varying levels of research understanding and variation in their understandings from passive recipients to active participants in research. By doing so, faculty can foster inquiry-driven learning environments that support students in developing a sense of scholarly identity and appreciating the relevance of research to their academic and professional futures.

## Supplementary Information

Supplementary Material 1.

## Data Availability

The datasets generated and analysed during the current study are not publicly available due to restrictions in the ethical approval.

## Abbreviations

COREQ: Consolidated Criteria for Reporting Qualitative Research
TLA: Teaching and Learning Activity
