# Supplementary material for: Unpacking the teaching–research nexus: a phenomenographic analysis of student experiences in a medical university
Source: BMC Med Educ. 2026 Oct 21;26:1690. doi: 10.1186/s12909-026-10653-5 (PMC13685128; doi:10.1186/s12909-026-10653-5)
Supplement: Supplementary file 1 [file 12909_2026_10653_MOESM1_ESM.docx]

**Interview guide**

1. Can you tell us a little about yourself, e.g. age, program, semester, previous university studies?
2. What is *research* to you?
3. In what ways can you take part in *research* during your education?
   a. Can you give some examples?
4. Is *research* discussed as part of your education?
   a. in teaching activities?
   b. in conversations between students and teachers?
   c. in conversations among students?
5. What thoughts do you have about the concept of research connection/basis?
   a. What do you think this concept means?
6. How do you think education should be connected to research?
   a. Can you give an example?
   b. One possible way is that education is delivered by research-active teachers.
   c. Another way is that the course literature includes research articles published in journals considered important by the research community.
   d. A third way is that teaching focuses on research results and research processes, for example by having students discuss scientific texts or studies.
7. How would you describe the overall degree of research connection/basis in the courses within your program?
8. Research and education can be located physically close to each other. Do you experience this in your program at KI?
   a. Have you had the opportunity to see research being conducted?
9. How do you feel that you are currently able to understand and value scientifically based knowledge?
   a. Do you use research when searching for information or studies?
      i. Have you learned it in education?
   b. Do you use research-based information from sources such as social media?
10. The very core of universities is the close link between teaching and research. However, some parts of education may rely more on proven experience and the needs of working life. Do you perceive any tension between these?
11. What do you think would help strengthen the research connection/basis in your courses or programme?
    a. Do you have any suggestions on how this could be done?
